# Intra- and extra-hospital improvement in ischemic stroke patients: influence of reperfusion therapy and molecular mechanisms

**DOI:** 10.1038/s41598-020-60216-x

**Published:** 2020-02-26

**Authors:** Pablo Hervella, Emilio Rodríguez-Castro, Manuel Rodríguez-Yáñez, Susana Arias, María Santamaría-Cadavid, Iria López-Dequidt, Ana Estany-Gestal, Elena Maqueda, Ignacio López-Loureiro, Tomás Sobrino, Francisco Campos, José Castillo, Ramón Iglesias-Rey

**Affiliations:** 1 Clinical Neurosciences Research Laboratory, Health Research Institute of Santiago de Compostela (IDIS), Hospital Clínico Universitario, Santiago de Compostela, Spain; 2 Stroke Unit, Department of Neurology, Hospital Clínico Universitario, Santiago de Compostela, Spain; 3 Unit of Methodology of the Research, Health Research Institute of Santiago de Compostela (IDIS), Santiago de Compostela, Spain

## Abstract

Neuroprotective treatments in ischemic stroke are focused to reduce the pernicious effect of excitotoxicity, oxidative stress and inflammation. However, those cellular and molecular mechanisms may also have beneficial effects, especially during the late stages of the ischemic stroke. The objective of this study was to investigate the relationship between the clinical improvement of ischemic stroke patients and the time-dependent excitotoxicity and inflammation. We included 4295 ischemic stroke patients in a retrospective study. The main outcomes were intra and extra-hospital improvement. High glutamate and IL-6 levels at 24 hours were associated with a worse intra-hospital improvement (OR:0.993, 95%CI: 0.990–0.996 and OR:0.990, 95%CI: 0.985–0.995). High glutamate and IL-6 levels at 24 hours were associated with better extra-hospital improvement (OR:1.13 95%CI, 1.07–1.12 and OR:1.14, 95%CI, 1.09–1.18). Effective reperfusion after recanalization showed the best clinical outcome. However, the long term recovery is less marked in patients with an effective reperfusion. The variations of glutamate and IL6 levels in the first 24 hours clearly showed a relationship between the molecular components of the ischemic cascade and the clinical outcome of patients. Our findings suggest that the rapid reperfusion after recanalization treatment blocks the molecular response to ischemia that is associated with restorative processes.

## Introduction

The ischemic cascade is a complex molecular and cellular process that is triggered immediately after an ischemic stroke (IS). During this process, several mechanisms are developed sequentially as a consequence of the deprivation of blood and oxygen supply to the ischemic core^1–7^. Neuroprotective treatments in acute IS are mainly focused to reduce the pernicious effect of excitotoxicity, oxidative-nitrosative stress, and inflammation^7,8^, however, these cellular and molecular mechanisms could also have reparative and beneficial effects for the patients. The reduction of glutamate concentration in the synaptic cleft has been the target of several clinical trials using antagonists of glutamate N-methyl-D-aspartate receptors to alleviate excitotoxicity, however those trials have failed to show clinical efficacy^8,9^. Targeting glutamate receptors can also block the synaptic transmission, and even though it could protect from glutamate excitotoxicity, it could also hinder the promotion of neuronal survival and cerebral plasticity in the late phase of IS^9^. Inflammation can also amplify the ischemic lesion and a considerable number of clinical trials were designed to target the inflammatory response^10^. Nonetheless, it is now clear that inflammation also promotes critical events necessary for tissue repair during the late stages of the IS^11^.

Recanalization treatments aimed to achieve reperfusion, either with the tissue Plasminogen Activator (tPA) or by mechanical thrombectomy, are the only treatments that have shown clinical benefit in IS, with a better clinical outcome observed in patients treated with recanalization therapies compared with untreated patients, both at short and long term^12–15^. Preclinical studies have shown that reperfusion could also modify the molecular profile of the ischemic cascade^1,16–18^, although the clinical implications of these findings are yet to be proven. In addition, reperfusion is associated with detrimental side effects. For instance, the potential toxic effect of tPA has been demonstrated in patients who did not reperfuse after the recanalization treatment^19,20^. Reperfusion is also associated with secondary damage due to oxidative stress^8,21^, although its repercussion on the clinical evolution of patients has not been demonstrated.

The clinical benefits of reperfusion are limited by several factors, including its narrow therapeutic window, the topography of the vascular occlusion and the type of thrombus, as well as by other factors related to the patients, such as age and the hemodynamic situations. The risks associated with reperfusion therapies require the best possible selection of patients amenable to recanalization therapies. Since recanalization treatment is known to modify the patient’s outcome, in this work we studied the improvement of patients in relation with the success of the recanalization treatment, both at short term or intra-hospital improvement (patient improvement between admission and discharge) and at long term or extra-hospital improvement (patient improvement between discharge and 3 months afterwards). We also evaluated the relation between patient improvement with the time-dependent excitotoxic and/or inflammatory mechanisms. The aim of the present study was to evaluate whether the levels of glutamate, IL6 and the reperfusion have a direct influence in the intra-hospital and extra-hospital improvement, in patients with IS.

## Results

The IS patients included in the analysis were classified into 3 groups: patients who did not receive recanalization treatment (PNR); patients with recanalization treatment and effective reperfusion (PER) and patients with recanalization treatment and uneffective reperfusion (PWER). The results are summarized in Table 1, including clinical and analytical variables.

**Table 1:** Demographic data, risk factors, baseline clinical characteristics, biochemical parameters, stroke subtype and type of thrombolytic treatment in Patients without recanalization treatment (PNR); patients with recanalization treatment and effective reperfusion (PER) and patients with recanalization treatment and uneffective reperfusion (PWER).

	PNR n = 3274	PER n = 342	PWER n = 679	p
Age, years	72.1 ± 13.9	71.2 ± 14.4	70.5 ± 13.5	0.044
Women, %	44.7	48.5	46.1	0.341
Onset-inclusion, min	263.9 ± 184.7	152.9 ± 52.8	187.3 ± 88.2	<0.0001
Previous mRS	0 [0, 1]	0 [0, 0]	0 [0, 0]	0.264
Stroke on awakening, %	10.9	3.8	7.5	<0.0001
History of hypertension, %	63.3	62.3	61.9	0.741
History of diabetes, %	25.0	17.3	22.8	0.004
History of smoking, %	16.3	15.8	18.3	0.424
History of enolism %	11.9	7.3	12.5	0.030
History of dyslipidemia, %	33.7	38.0	38.9	0.017
History of atrial fibrillation, %	19.4	19.0	21.1	0.583
History of ischemic heart disease, %	11.0	8.5	14.3	0.011
History of heart failure, %	4.0	4.1	4.7	0.674
History of carotid disease, %	1.1	0.3	0.9	0.342
Previous TIA, %	5.5	5.8	4.7	0.651
Axillary temperature on admission, °C	36.3 ± 0.6	36.4 ± 0.5	36.5 ± 0.7	<0.0001
Glycemia, mg/dL	136.9 ± 59.2	123.6 ± 34.1	136.7 ± 51.1	<0.0001
Leukocytes, x10^3^/mL	8.7 ± 2.6	8.6 ± 3.8	10.2 ± 3.1	<0.0001
Fibrinogen, mg/dL	438.1 ± 83.0	437.7 ± 119.2	433.2 ± 109.1	<0.0001
Microalbuminuria, mg/24 h	8.6 ± 28.3	4.2 ± 5.6	5.8 ± 12.3	0.578
Reactive protein C, mg/L	2.2 ± 3.3	4.1 ± 3.8	4.4 ± 4.2	<0.0001
Glycosylated hemoglobin, %	6.2 ± 1.5	5.7 ± 0.5	6.2 ± 1.7	0.040
LDL cholesterol, mg/dL	118.1 ± 39.6	116.9 ± 28.9	112.3 ± 40.5	0.063
HDL cholesterol, mg/dL	41.7 ± 16.5	37.2 ± 9.4	43.1 ± 28.7	0.939
Triglycerides, mg/dL	122.6 ± 71.5	124.6 ± 41.9	134.2 ± 63.9	0.011
Sedimentation rate, mm	18.9 ± 16.4	18.9 ± 27.7	21.4 ± 22.9	0.010
NT-proBNP, pg/mL	539.4 ± 1132.2	620.4 ± 1467.3	639.0 ± 934.3	0.037
Intima-media thickness, mm	0.9 ± 0.8	0.9 ± 0.2	0.8 ± 0.2	0.355
NIHSS on admission	13 [10,20]	18 [15,22]	14 [9,20]	<0.0001
NIHSS at 24 hours	12 [6,18]	4 [2,8]	15 [8,21]	<0.0001
Early neurological improvement (%)	24.8	100	0.2	<0.0001
NIHSS at 48 hours	8 [4,14]	3 [0, 7]	12 [7,12]	<0.0001
NIHSS on discharge	6 [3,12]	1 [0, 4]	12 [10,16]	<0.0001
NIHSS at 3 months	2 [2, 2]	0 [0, 0]	9 [6,15]	<0.0001
mRS on discharge	3 [2,4]	2 [1,3]	3 [2,4]	<0.0001
mRS at 3 months	2 [1,3]	0 [0, 1]	1 [0, 3]	<0.0001
Infarct volume (CT 4th-7th day), mL	38.1 ± 77.4	24.4 ± 30.8	35.4 ± 53.6	<0.0001
Hemorrhagic transformation, %	9.5	21.6	14.3	<0.0001
TOAST				<0.0001
- Atherothrombotic, %	22.8	19.6	31.1	
- Cardioembolic, %	34.8	45.0	37.6	
- Lacunar, %	10.5	1.8	1.2	
- Undetermined, %	30.6	33.0	29.4	
- Others, %	1.3	0.6	0.7	
Type of reperfusion treatment, %				0.059
- Intravenous tPA, %	—	79.2	82.5	
- Thrombectomy, %	—	14.1	13.5	
- Mixed, %	—	6.7	4.0	
Intra-hospital improvement, %	9.1	45.9	3.9	<0.0001
Extra-hospital improvement, %	11.7	0.5	5.0	<0.0001

CT: Computed Tomography; mRS: modified Rankin Scale; NIHSS: National institute of Health Stroke Scale; NT-proBNP: N-terminal pro b-type Natriuretic Peptide; TIA: Transient ischemic attack; TOAST: Trial of Org 10172 in Acute Stroke Treatment; tPA: Tissue Plasmingen Activator.

The selection criteria of patients for recanalization partially determined the statistical differences between the groups. In this regard, PER showed the highest clinical improvement, and the benefit persists during the first three months after the treatment (mRS at 3 months: 0[0,1] for PER vs 2[1,3] for PNR and 1[0,3] for PWER. p < 0.0001).

The NIHSS at hospital discharge was significantly higher for PWER compared to PNR and PER (12[10,16], 6[3,12] and 1[0,4]. p < 0.0001). Regarding the intra-hospital improvement, it was much higher for PER compared with PNR and PWER (45.9 ± 12.8, 9.1 ± 10.1 and 3.9 ± 17.2, p < 0.0001). However, this trend was different for the extra-hospital improvement, with the highest values observed in PNR, followed by PWER and PER (11.7 ± 10.8, 5.0 ± 8.3 and 0.5 ± 2.9, p < 0.0001).

### Intra-hospital improvement

Intra-hospital improvement was defined as a positive value of the NIHSS scale on admission minus the value of the NIHSS scale at discharge. Among the total sample (4295 patients), 1176 patients (27.4%) did not show any improvement during hospitalization, including 398 deaths, while 3119 patients (72.6%) showed an improvement of at least 1 point in the NIHSS between admission and discharge. It can be seen (Table 2) that intra-hospital improvement is a good marker for good outcome at 3 months, defined as mRS values ≤2 (59.6% for patients with intra-hospital improvement and 31.9% for patients without intra-hospital improvement, p < 0.0001). Intra-hospital improvement was also related with lower values of markers associated with acute inflammatory response, such as temperature (36.3 ± 0.6 °C vs 36.5 ± 0.7 °C, p < 0.0001), leukocytes (9.2 ± 3.1 × 10^3^ mL vs 9.7 ± 3.1×10^3^ mL, p < 0.0001), fibrinogen (444.7 ± 95.5 mg/dL vs 447.0 ± 104.2 mg/dL, p = 0.004), C-reactive protein (2.8 ± 3.5 mg/L vs 3.9 ± 4.5 mg/L, p < 0.0001) and sedimentation rate (25.4 ± 22.6 mm vs 25.7 ± 23.0 mm, p = 0.003).

**Table 2:** Clinical characteristics, biochemical parameters, stroke subtype and type of thrombolytic treatment for patients with and without intra-hospital improvement.

	NO n = 1176	YES n = 3119	p
Age, years	72.9 ± 13.5	71.3 ± 14.0	<0.0001
Women, %	47.7	44.2	0.023
Onset-inclusion, min	239.9 ± 158.5	239.7 ± 171.0	0.965
Previous mRS	0 [0, 1]	0 [0, 1]	0.722
Stroke on awakening, %	10.1	9.7	0.353
History of hypertension, %	65.1	62.2	0.047
History of diabetes, %	23.7	24.2	0.387
History of smoking, %	15.3	17.1	0.091
History of enolism %	11.8	11.6	0.442
History of dyslipidemia, %	36.7	34.2	0.063
History of atrial fibrillation, %	23.3	18.2	<0.0001
History of ischemic heart disease, %	13.8	10.4	0.001
History of heart failure, %	4.8	3.8	0.077
History of carotid disease, %	0.8	1.1	0.220
Previous TIA, %	5.5	5.4	0.454
Axillary temperature on admission, °C	36.5 ± 0.7	36.3 ± 0.6	<0.0001
Glycemia, mg/dL	143.7 ± 66.0	136.5 ± 54.9	<0.0001
Leukocytes, × 10^3^/mL	9.7 ± 3.1	9.2 ± 3.1	<0.0001
Fibrinogen, mg/dL	447.0 ± 104.2	444.7 ± 95.5	0.004
Microalbuminuria, mg/24 h	8.7 ± 25.2	8.3 ± 27.3	0.740
Reactive protein C, mg/L	3.9 ± 4.5	2.8 ± 3.5	<0.0001
Glycosylated hemoglobin, %	6.1 ± 1.4	6.1 ± 1.3	0.756
LDL cholesterol, mg/dL	108.0 ± 37.3	112.3 ± 37.7	0.015
HDL cholesterol, mg/dL	41.8 ± 14.3	41.5 ± 18.7	0.231
Triglycerides, mg/dL	112.5 ± 56.8	120.0 ± 62.7	0.539
Sedimentation rate, mm	25.7 ± 23.0	25.4 ± 22.6	0.003
NT-proBNP, pg/mL	1821.9 ± 2130.4	1175.7 ± 1883.1	<0.0001
Intima-media thickness, mm	0.9 ± 0.5	1.0 ± 0.7	0.355
Glutamate on admission	234.5± 121.1	192.7± 121.5	<0.0001
Glutamate at 24 hours	165.4±74.6	131.9± 80.0	<0.0001
IL6 on admission	21.1±15.8	21.3±15.2	0.844
IL6 at 24 hours	62.2± 47.5	51.1±46.3	0.006
Infarct volume (CT 4th-7th day), mL	41.8 ± 75.6	24.6 ± 52.2	<0.0001
Hemorrhagic transformation, %	13.9	10.6	0.004
TOAST			<0.0001
- Atherothrombotic, %	25.3	23.3	
- Cardioembolic, %	41.8	34.0	
- Lacunar, %	3.4	10.1	
- Undetermined, %	29.1	31.2	
- Others, %	0.4	1.4	
Study group			<0.0001
- Not recanalization (PNR), %	66.5	79.9	
- Effective reperfusion (PER), %	0.2	10.9	
- Without effective reperfusion (PWER), %	33.3	9.2	
Type of reperfusion treatment, %			0.064
- Intravenous tPA, %	83.2	80.8	
- Thrombectomy, %	12.8	13.5	
- Mixed, %	4.0	5.7	
NIHSS on admission	15 [8,21]	14 [8,19]	0.056
NIHSS at 3 months	6 [2,12]	2 [2, 2]	<0.0001
Intrahospital Improvement	−2.99±7.0	17.9±14.6	<0.0001
Extrahospital Improvement	11.55±10.1	9.67±10.7	<0.0001
mRS at 3 months	3 [1,4]	2 [0, 3]	<0.0001
Good outcome at 3 months, %	31.9	59.6	<0.0001

CT: Computed Tomography; mRS: modified Rankin Scale; NIHSS: National institute of Health Stroke Scale; NT-proBNP: N-terminal pro b-type Natriuretic Peptide; TIA: Transient ischemic attack; TOAST: Trial of Org 10172 in Acute Stroke Treatment; tPA: Tissue Plasmingen Activator.

The type of recanalization treatment did not influence the intra-hospital improvement (Table S1, p = 0.064), but intra-hospital improvement was more frequent in PER compared with PWER (10.9% vs 9.2%, p < 0.0001).

The data was also analyzed in a logistic regression model (Table S1), using the intra-hospital improvement as the dependent variable and the relationship of effective reperfusion therapy with intra-hospital improvement was confirmed (OR: 38.13, 95%CI: 5.26–276.64; p < 0.0001). Likewise, an ineffective reperfusion therapy was associated with a negative intra-hospital evolution (OR: 0.26, 95%CI: 0.19–0.35; p < 0.0001).

### Extra-hospital improvement

Extra-hospital improvement was defined as a positive value of the NIHSS scale at discharge minus the value of the NIHSS scale at three months. Extra-hospital improvement was analyzed in 3853 patients (Table 3). Among those patients, 1326 (34.4%) did not show extra-hospital improvement while 2527 (65.6%) showed an improvement of at least 1 point in the NIHSS 3 ± 1 months after the hospital discharge. A bivariate analysis was carried out classifying the patients into two groups depending on the presence or absence of extra-hospital improvement (Table 3), and it was observed that the extra-hospital improvement was not related with a good outcome at 3 months, defined as mRS values ≤2 (64.9% vs 60.5%, p = 0.051). Extra-hospital improvement was associated with higher values of inflammatory markers (leukocytes 8.7 ± 3.1×10^3^ mL vs 9.4 ± 3.1 × 10^3^ mL, p = 0.001; fibrinogen 437.6 ± 94.9 mg/dL vs 445.3 ± 98.6 mg/dL, p = 0.002; and sedimentation rate 23.2 ± 23.6 mm vs 26.5 ± 22.8 mm, p < 0.0001). Also, extra-hospital improvement was higher in patients with more serious infarcts (infarct volume 40.8 ± 69.1 mL vs 11.2 ± 31.3 mL, p < 0.0001). The type of reperfusion treatment did not influence the extra-hospital improvement, however, it was less frequent in PER (2.7%) compared with PNR (79.7%) and with PWER (17.6%).

**Table 3:** Clinical characteristics, biochemical parameters, stroke subtype and type of thrombolytic treatment for patients with and without extra-hospital improvement.

	NO n = 1326	YES n = 2527	p
Age, years	71.8 ± 13.3	70.1 ± 12.2	<0.0001
Women, %	41.7	44.8	0.070
Onset-inclusion, min	240.3 ± 195.7	228.9 ± 139.1	0.832
Previous mRS	0 [0, 1]	0 [0, 1]	0.162
Stroke on awakening, %	10.6	7.4	0.001
History of hypertension, %	62.9	61.7	0.485
History of diabetes, %	24.4	23.9	0.751
History of smoking, %	17.7	17.1	0.662
History of enolism %	13.3	8.7	<0.0001
History of dyslipidemia, %	35.7	34.5	0.445
History of atrial fibrillation, %	19.9	14.0	<0.0001
History of ischemic heart disease, %	11.3	10.1	0.276
History of heart failure, %	3.9	3.5	0.592
History of carotid disease, %	1.1	1.0	0.744
Previous TIA, %	7.1	4.8	0.004
Axillary temperature on admission, °C	36.3 ± 0.6	36.3 ± 0.6	0.078
Glycemia, mg/dL	141.1 ± 60.9	130.1 ± 47.2	<0.0001
Leukocytes, × 10^3^/mL	8.7 ± 2.9	9.4 ± 3.2	0.001
Fibrinogen, mg/dL	437.6 ± 94.9	445.3 ± 98.6	0.002
Microalbuminuria, mg/24 h	12.0 ± 37.2	7.4 ± 22.4	0.656
Reactive protein C, mg/L	2.7 ± 3.8	3.1 ± 3.6	<0.0001
Glycosylated hemoglobin, %	6.0 ± 1.2	6.1 ± 1.3	0.482
LDL cholesterol, mg/dL	113.4 ± 36.8	109.7 ± 37.5	0.105
HDL cholesterol, mg/dL	39.9 ± 15.2	42.5 ± 19.6	0.030
Triglycerides, mg/dL	124.0 ± 63.9	115.7 ± 58.2	<0.0001
Sedimentation rate, mm	23.2 ± 23.6	26.5 ± 22.8	<0.0001
NT-proBNP, pg/mL	1378.4 ± 1937.4	797.1 ± 1435.2	<0.0001
Intima-media thickness, mm	0.9 ± 0.6	0.9 ± 0.7	0.268
Glutamate on admission	194.2 ± 116.6	221.1 ± 125.8	0.002
Glutamate at 24 hours	80.3 ± 36.7	219.6 ± 43.2	<0.0001
IL6 on admission	22.4 ± 15.3	20.1 ± 15.3	0.043
IL6 at 24 hours	20.1 ± 25.0	96.5 ± 30.8	<0.0001
Infarct volume (4th-7th day), mL	11.2 ± 31.3	40.8 ± 69.1	<0.0001
Hemorrhagic transformation, %	11.2	7.9	0.005
TOAST			0.077
- Atherothrombotic, %	29.9	27.9	
- Cardioembolic, %	28.0	30.3	
- Lacunar, %	11.0	8.3	
- Undetermined, %	29.9	32.2	
- Others, %	1.1	1.3	
Study group			<0.0001
- Not recanalized (PNR), %	69.4	79.7	
- Effective reperfusion (PER), %	20.5	2.7	
- Without effective reperfusion (PWER), %	10.1	17.6	
Type of reperfusion treatment, %			0.164
- Intravenous tPA, %	84.1	83.7	
- Thrombectomy, %	13.2	13.1	
- Mixed, %	2.7	3.2	
NIHSS on admission	5 [2,9]	13 [9,17]	<0.0001
NIHSS at 3 months	2 [1,4]	2 [1,5]	0.051
Intrahospital Improvement	19.5 ± 18.8	8.4 ± 13.1	<0.0001
Extrahospital Improvement	−1.25 ± 3.4	15.5 ± 8.3	<0.0001
mRS at 3 months	0 [0, 3]	0 [0, 4]	0.134
Good outcome at 3 months, %	64.9	60.5	0.078

CT: Computed Tomography; mRS: modified Rankin Scale; NIHSS: National institute of Health Stroke Scale; NT-proBNP: N-terminal pro b-type Natriuretic Peptide; TIA: Transient ischemic attack; TOAST: Trial of Org 10172 in Acute Stroke Treatment; tPA: Tissue Plasmingen Activator.

A logistic regression analysis was carried out using the extra-hospital improvement as a dependent variable (Table S2), showing that extra-hospital improvement is significantly negative in PER (OR:0.02, 95%CI: 0.01–0.03, p < 0.0001).

### Molecular mechanisms (excitotoxicity and inflammation) associated with intra and extra-hospital improvement

**Figure 1:**
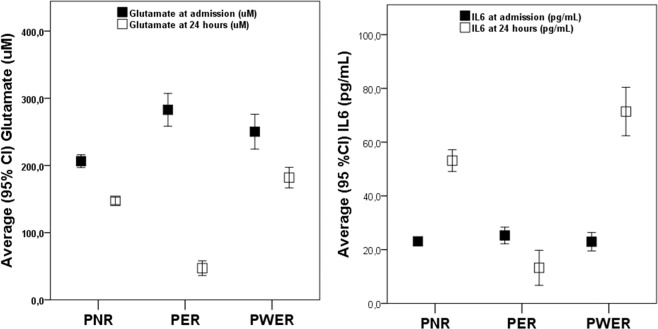
Mean concentrations of Glutamate (left) and IL6 (right) concentrations on admission and at 24 hours in PER, PNR and PWER.

In order to evaluate the influence of excitotoxicity and inflammation on the intra and extra-hospital improvement, an analysis of glutamate and IL6 levels was carried out in patients on admission and after 24 h (Table S3). Serum glutamate levels decreased significantly in the first 24 hours for the three groups of patients, although this decrease was more pronounced in PER (Fig. 1). The difference between the glutamate levels on admission in the three groups of patients is attributed to the selection criteria of patients for reperfusion treatments, the onset-inclusion time and the severity of the neurological deficit (both listed in Table S3), demonstrated by the correlations between the NIHSS and the glutamate levels on admission in the three groups of patients.

Serum IL6 levels on admission were similar for the three groups of patients, however, IL6 levels were significantly higher in PNR and PWER at 24 hours compared with PER. Moreover, only PER showed a decrease in IL6 levels after 24 h.

Glutamate levels on admission were related with a worse intra-hospital evolution (OR:0.996, 95%CI: 0.995–0.998, p < 0.0001); however, this relationship was not demonstrated for basal IL-6 levels (OR:1.005, 95%CI: 0.996–1.0014, p = 0.292). After 24 hours, both glutamate and IL6 levels correlated with a worse intra-hospital evolution (OR:0.993, 95%CI: 0.990–0.996, p < 0.0001 and OR:0.990, 95%CI: 0.985–0.995, p < 0.0001 respectivelly) (Table S4). These results are consistent with a large number of publications that justify the use of drugs to reduce excessive excitotoxic and inflammatory response in IS^22,23^.

Surprisingly, and opposed to the trend observed for intra-hospital improvement, the extra-hospital improvement (Table S5) was associated with higher glutamate and IL6 levels determined at 24 h (OR:1.132, 95%CI, 1.072–1.196, p < 0.0001 and OR:1.137, 95%CI, 1.094–1.183, p < 0.0001).

The association of glutamate and IL6 levels with intra and extra-hospital improvement was further confirmed using a multiple linear regression analysis (Table S6). Glutamate levels on admission were correlated with positive intra and extrahospital improvement (β:0.025, p = 0.017 and β:0.019, p = 0.002 respectively), however, there was a negative correlation of high glutamate levels at 24 hours and intrahospital improvement (β −0.042, p = 0.046). Regarding IL6, high levels of this marker on admission had a negative correlation with extrahospital improvement (β −0.305, p < 0.0001), while high levels of IL6 at 24 hours correlates with a negative intrahospital improvement and a positive extrahospital improvement (β: −0.121, p < 0.0001 vs β: 0.131, p < 0.0001). These analyses showed the association between glutamate and IL6 and are further discussed below.

## Discussion

To the best of our knowledge, this is the first study aimed to analyse the early (intra-hospital) and late (extra-hospital) evolutionary outcome of IS patients in relation to the recanalization treatment.

Recanalization treatments have demonstrated high survival rates and good outcome in clinical trials^13–15^. In our study, we also observed the best clinical outcome in patients with effective reperfusion as a response to the most appropriate recanalizing treatment, both at the time of hospital discharge and after 3 months. Indeed, the multivariate analysis demonstrates that early improvement is 38 times more frequent in patients with effective reperfusion compared with patients without recanalization treatment. Patients who received an uneffective recanalization treatment were the ones with the worst clinical situation. These results are in agreement with other clinical studies, as it is now recognized that effective reperfusion is an accurate indicator for clinical outcome in IS patients^24,25^.

**Figure 2:**
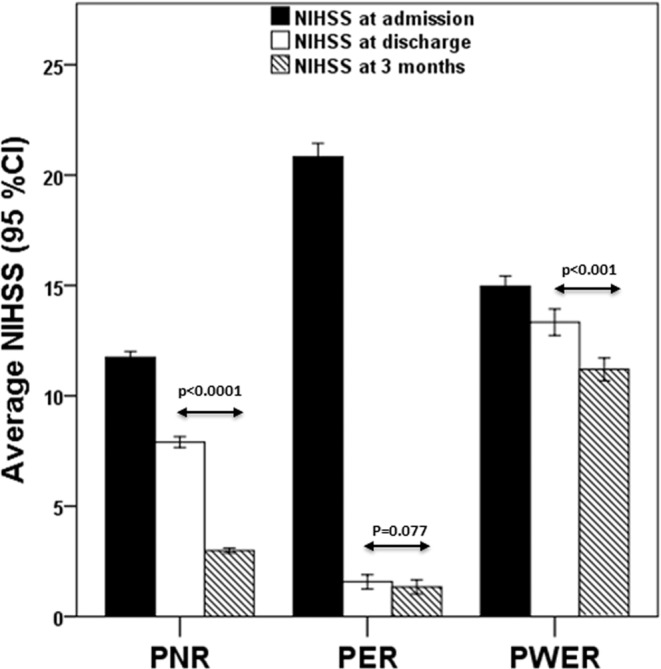
Variation of the NIHSS on admission, at discharge and 3 months after hospitalization in PER, PNR and PWER.

The main factors independently associated with a negative intra-hospital evolution are hyperthermia, infarct volume and especially the uneffective reperfusion. However, effective reperfusion is related to worst extra-hospital improvement. Logically, the extra-hospital evolution is greater in patients who present a higher NIHSS at discharge, and therefore, the margin for improvement is lower for PER (Fig. 2). However, we hypothesize that the effective early reperfusion could also hinder neuroprotective molecular mechanisms. Although this hypothesis cannot be demonstrated in this study, the variations in glutamate and IL6 levels in the first 24 hours clearly show a relationship between the molecular components of the ischemic cascade and the clinical outcome of patients after 3 months, as discussed next.

**Figure 3:**
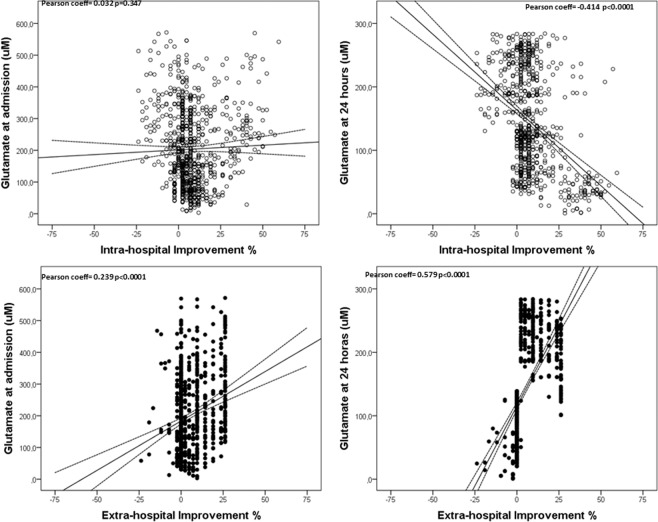
Correlation between the glutamate levels on admission and after 24 hours and the intra-hospital and extra-hospital improvement.

Serum glutamate levels on admission are related to the stroke severity, and high levels of glutamate at and 24 h after admission were independently associated with a worse intra-hospital evolution. This is in agreement with previous studies^26,27^, although the clinical significance of these findings is rather low taking into account the nature of these variables and its units. Surprisingly, we observed an association between high glutamate levels at 24 hours and better extra-hospital evolution, and we determined that high glutamate levels at 24 hours are independently associated with an 11% better extra-hospital improvement, with both statistical and clinical significance. These observations suggested that the intense reduction of glutamate in the first 24 hours may be a factor that leads to a lower late recovery after an effective reperfusion (Fig. 3).

**Figure 4:**
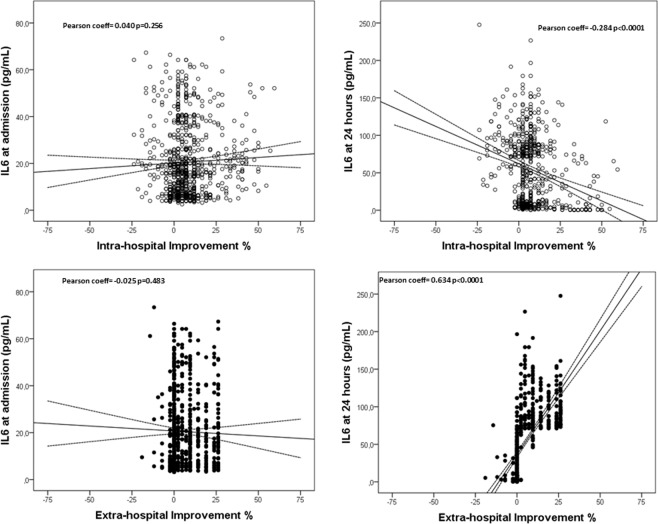
Correlation between the IL6 levels on admission and after 24 hours and the intra-hospital and extra-hospital improvement.

The inflammatory response has also been associated with early clinical deterioration^28,29^, and a worse prognosis after IS^30^. In our study, the inflammatory response was similar in the three groups of patients at admission; however we found significant differences on IL6 levels in serum at 24 h. IL6 levels significantly increased after 24 hours in PNR and PWER, but decreased in PER. Similar to the trend observed with glutamate, high levels of IL6 at 24 hours are independently associated with a worse intra-hospital evolution, but also with an 11% increase in extra-hospital improvement. A reduction in IL6 levels after 24 hours is another factor correlated with a worse extra-hospital improvement in IS patients (Fig. 4).

This is a retrospective and single-center study. However, the study comprises a large and unbiased sample (5306 patients) for the purposes of this analysis. The use of a clinical criterion to define effective reperfusion could lead to a bias in the classification in therapeutic response groups. The Doppler and MR angiography study after recanalization treatment was limited to a small group of patients. The narrow therapeutic margin available for patients with good therapeutic response could reduce the benefits in this group of patients; however, to avoid this effect we used the percentage deviation, not absolute, between the score of the NIHSS at discharge and at 3 months. The 3-month follow-up time is probably too short to evaluate the long-term effects. We have made an analysis in a subgroup of 287 patients that we have followed for a year, and the results were similar (data not shown).

## Conclusions

Effective reperfusion after recanalization treatments provided the best clinical outcome for IS patients. However, this clinical improvement is usually achieved at an early stage, and long term recovery is less marked in PER. It is likely that the rapid reperfusion blocks the molecular response to ischemia, which is associated with restorative processes and cerebral plasticity, and some degree of excitatory and inflammatory mechanisms may be associated with the late recovery of patients.

The results of this work may involve the following practical suggestions: treatments targeting excitotoxicity may be optimal if administered in very short therapeutic windows and the inhibition of excitotoxicity after the first hours could block molecular responses for brain healing. The use of peripheral glutamate grabbers, which do not affect the mechanisms of the excitatory response in the brain, could be therefore an interesting therapeutic alternative^22^. The administration of anti-inflammatory drugs in large therapeutic windows could have negative effects. In this sense, the use of inflammatory markers like IL6 could be used not only for diagnosis but also as neuroprotectors in the management of IS^31,32^.

## Methods and Patients

### Study design

This is a retrospective analysis conducted on a prospective registry of patients with acute cerebrovascular disease consecutively admitted to the Stroke Unit of the University Clinical Hospital of Santiago de Compostela (Spain) from September 2007 to March 2018. We prospectively included all IS patients admitted to the Stroke Unit in the BICHUS registry. The study was carried out in accordance with the Declaration of Helsinki of the World Medical Association and approved by the Research Ethics Committee of Galicia. Written informed consent was obtained on admission from each patient or from their relatives after full explanation of the procedures.

The clinical questions addressed in this study expressed in PICO (Patient, Intervention, Comparison, Outcome) format were:

Among IS patients, do high levels of glutamate, IL6 and effective reperfusion compared with low levels of the mentioned makers and with uneffective reperfusion and non recanalization treatments improve the functional outcome at short and long term after hospitalization?

### Clinical variables

All patients were handled by the same stroke team according to the Stroke Unit protocol. The variables of our registry included the date, onset-inclusion and wake-up strokes. Moreover, demographic data, previous modified Rankin scale score (mRS) and vascular risk factors were included. Data on previous carotid disease and processes of carotid revascularization were also collected, and in patients with a previous transient ischemic attack (TIA), the time between the TIA and stroke, as well as the coincidence or not with the topography. Medical history recording potential vascular risk factors, blood and coagulation test, 12-lead ECG and chest x-ray were collected. Carotid and transcranial Doppler were performed on admission; the transcranial Doppler study was repeated as a control of the recanalization treatment in 18% of the treated patients. Axillary temperature was obtained on admission and every 4 hours during the first 48 hours. The outcome clinical variables recorded were: National Institute of Health Stroke Scale (NIHSS), an internationally validated scale used to quantify the neurological impairment caused by a stroke, with values ranging from 0 (no stroke symptoms) to 42 (severe stroke), measured on admission, 24 hours, 48 hours, at discharge and at 3 ± 1 months; and the modified Ranking Scale (mRS), which is commonly used for measuring the degree of disability or dependence in the activities of stroke patients, ranging from 0 (no symptoms) to 6 (dead), measured at discharge and at 3 ± 1 months. Good outcome was considered for patients with a mRS value of 0,1 and 2. Both scales were performed by accredited neurologist.

On the other hand, IS stroke subtypes were classified according to the TOAST criteria and hemorrhagic strokes were classified as hypertensive, amyloid or related to antiplatelet/anticoagulant. The type of reperfusion treatment, its characteristics and complications were also recorded.

At discharge, 28.7% of the patients were institutionalized and the rest were reintegrated into their family environment. During hospitalization, candidate patients for rehabilitation were evaluated by the Rehabilitation Service according to a common protocol. The outpatient rehabilitation was coordinated by the primary care physician, but it was not protocolized in an even way.

### Analytical determinations

Biochemistry, haematology and coagulation test were assessed in the central laboratory of the hospital. For the molecular determinations, venous blood samples were collected in Vacutainer tubes (Becton Dickinson, San Jose, CA, USA) on admission, and 24 ± 12 hours and/or 48 ± 12 hours from stroke onset. After clotting for 60 minutes, blood samples were centrifuged at 3000 × g for 10 minutes, and the serum was immediately aliquoted, frozen and stored at −80 °C until analysis.

Glucose levels, glycosylated hemoglobin, leukocytes, red blood cells, platelets, fibrinogen, C-reactive protein, total and fractionated cholesterol, triglycerides, proBNP, vitamin D and cholecalciferol were measured on admission. Serum levels of glutamate were determined on admission and after 24 hours by high performance liquid chromatography (HPLC) analysis (1260 Infinity II; Agilent Technologies) using the AccQ‐Tag derivatization method for amino acid analysis following the manufacturer´s technical specifications (Waters)^33^. Serum levels of interleukin 6 (IL6) were also measured on admission and after 24 hours using an immunodiagnostic IMMULITE 1000 System (Diagnostic Products Corporation, California, USA).

### Neuroimaging studies

All patients underwent cerebral computed tomography (CT) on admission and between days 4 and 7 after IS. Multimodal magnetic resonance imaging (MRI) was also performed on admission in IS patients candidates to recanalization treatments. Lesion volumes were measured using ABC/2 method^34^ in DWI-MRI on admission and betwen the 4^th^ and 7^th^ day in IS patients. In 2012, recanalization by endovascular treatment was protocolized in our hospital. Since 2012, a MRI angiography is performed in all patients candidate for reperfusion treatments. Neuroradiologists blinded to clinical and analytical data performed all imaging studies.

### Patients

We included 6565 stroke patients. Excluded patients were: ICH (1011), transient ischemic attack patients (512), patients transferred to other hospital during the acute phase (44), dead by other causes (40), no final stroke diagnosis (85), admitted outside the acute phase (97) and lacked follow-up at 3 months (480). Finally, 4295 patients were valid for the analysis.

Early neurological improvement (the reduction ≥8 points in the NIHSS in the first 24 hours^35^) was used as a clinical marker of effective reperfusion.

### Outcome endpoints

We have defined the intra-hospital improvement as the normalized percentage difference between the basal and discharge NIHSS through the formula: [(NIHSS on admission-NIHSS at discharge)/42 × 100], and the extra-hospital improvement between the NIHSS at discharge and at 3 ± 1 months, using the formula [(NIHSS at discharge-NIHSS at 3 months)/42 × 100] (42 was used to normalize the values as the maximum variation of the NIHSS).

We categorized the intra-hospital and extra-hospital improvement as a decrease ≥1 in the NIHSS between admission and discharge and between discharge and at 3 months, respectively. The deceased patients were given the highest NIHSS value.

### Statistical analysis

Firstly, an analysis was performed to describe the sample. Frequencies and percentages were calculated for categorical variables while mean and standard deviation [SD] or median and interquartile range were used for continuous variables depending on their adjustment to normality. Kolmogorov-Smirnov test with Lilliefors was applied to assess it. ANOVA and squared test were performed to determine differences in patients according with the reperfusion group (PNR, PER, PWER). The main objective of the study was to evaluate the intra and extra hospital improvement. In order to achieve it, multivariable models were built using a lineal regression analysis. The dependent variables were adjusted by clinical significant variables calculated in the bivariate analysis. Also, logistic regression analyses were carried out for patients with the same dependent variables but categorized. These results were shown as odds ratios (ORs) with 95% confidence intervals (CI 95%). P-value <0.05 was considered to be statistically significant. The statistical analyses were conducted in SPSS 21.0 (IBM, USA).

## Supplementary information

Supplemental Information.

## Data Availability

All data are available within the text of the manuscript. Further anonymized data could be made available to qualified investigators upon reasonable request.
