# Supplementary material for: Intra- and extra-hospital improvement in ischemic stroke patients: influence of reperfusion therapy and molecular mechanisms
Source: Sci Rep. 2020 Feb 26;10:3513. doi: 10.1038/s41598-020-60216-x (PMC7044227; doi:10.1038/s41598-020-60216-x)
Supplement: Supplementary file 1 — Supplemental Information. [file 41598_2020_60216_MOESM1_ESM.docx]

**Supplementary Information**

**Intra- and extra-hospital improvement in ischemic stroke patients: influence of reperfusion therapy and molecular mechanisms**

Pablo Hervella PhD^1^, Emilio Rodríguez-Castro MD PhD^1,2^, Manuel Rodríguez-Yáñez MD PhD^1,2^, Susana Arias MD PhD^1,2^, María Santamaría-Cadavid MD PhD^1,2^, Iria López-Dequidt MD^1,2^, Ana Estany-Gestal PhD^3^, Elena Maqueda BSc^1^, Ignacio López-Loureiro BSc^1^, Tomás Sobrino PhD^1^, Francisco Campos PhD^1^, José Castillo MD PhD^1^, Ramón Iglesias-Rey PhD^1^.

^1^Clinical Neurosciences Research Laboratory, Health Research Institute of Santiago de Compostela (IDIS), Hospital Clínico Universitario, Santiago de Compostela, Spain.

^2^Stroke Unit, Department of Neurology, Hospital Clínico Universitario, Santiago de Compostela, Spain.

^3^Unit of Methodology of the Research, Health Research Institute of Santiago de Compostela (IDIS), Spain.

Corresponding authors

Dr. Pablo Hervella and Dr. Ramón Iglesias-Rey

pablo.hervella.lorenzo@sergas.es, [ramón.iglesias.rey@sergas.es](mailto:ramón.iglesias.rey@sergas.es)

Telephone: +34 981951086

Fax number: +34 981951098

Table S1 Logistic regression analysis for intra-hospital improvement

| **Independent variables** | **OR*** | **CI 95%** | **p** |
| --- | --- | --- | --- |
| **Axillary temperature on admission** | 0.81 | 0.66 - 0.99 | 0.042 |
| **Infarct volume (CT 4th-7th day)** | 0.99 | 0.99 - 0.99 | <0.0001 |
| **Study group** |  |  |  |
| **- Patients with no recanalization (PNR)** | - | - | - |
| **- Effective reperfusion (PER)** | 38.13 | 5.26 - 276.64 | <0.0001 |
| **- Without effective reperfusion (PWER)** | 0.26 | 0.19 - 0.35 | <0.0001 |

** Adjusted by: Age, sex, history of hypertension, atrial fibrillation, ischemic heart disease, glycemia, leukocytes, fibrinogen, C-reactive protein, LDL-cholesterol, sedimentation rate, proBNP, hemorrhagic transformation and TOAST.*

**Table S2** Logistic regression analysis for extra-hospital improvement

| **Independent variables** | **OR*** | **CI 95%** | **p** |
| --- | --- | --- | --- |
| **NIHSS on admission** | 1.22 | 1.17 - 1.26 | <0.0001 |
| **Study group** |  |  |  |
| **- Patients with no recanalization (PNR)** | - | - | - |
| **- Effective reperfusion (PER)** | 0.02 | 0.01 - 0.03 | <0.0001 |
| **- Without effective reperfusion (PWER)** | 0.74 | 0.49 - 1.13 | 0.167 |

** Adjusted by: Age, stroke on awakening, enolism, atrial fibrillation, previous TIA, glycemia, leukocytes, fibrinogen, C-reactive protein, HDL-cholesterol, triglycerides, sedimentation rate, pro BNP, infarct volume and hemorrhagic transformation.*

**Table S3** Glutamate and IL6 concentrations at and 24 hours after admission; NIHSS and inclusion time.

|  | **PNR** | **PER** | **PWER** | **p** |
| --- | --- | --- | --- | --- |
| **Glutamate on admission, μM/mL** | 212.5 ± 123.1 | 294.2 ± 109.3 | 246.1 ± 128.0 | <0.0001 |
| **Glutamate at 24 h, μM/mL** | 146.5 ± 69.7 | 57.6 ± 52.5 | 186.4 ± 75.9 | <0.0001 |
|  | <0.0001 | <0.0001 | <0.0001 |  |
|  |  |  |  |  |
| **IL6 on admission, pg/mL** | 23.1 ± 15.9 | 25.3 ± 10.6 | 22.9 ± 15.7 | 0.134 |
| **IL6 at 24 h, pg/mL** | 53.1 ± 46.0 | 13.2 ± 22.5 | 70.7 ± 41.5 | <0.0001 |
|  | <0.0001 | <0.0001 | <0.0001 |  |
|  |  |  |  |  |
| **NIHSS on admission** | 13 [10, 20] | 18 [15, 22] | 14 [9, 20] | <0.0001 |
| **Onset-inclusion time, min** | 263.9 ± 184.7 | 152.9 ± 52.8 | 187.3 ± 88.2 | <0.0001 |

Table S4 Logistic regression analysis for intra-hospital improvement related with Glutamate and IL6 serum levels on admission and at 24 hours.

|  | **OR*** | **CI 95%** | **p** | **OR**** | **CI 95%** | **p** |
| --- | --- | --- | --- | --- | --- | --- |
| **Glutamate on admission** | 0.99 | 0.99 - 0.99 | <0.0001 | 0.996 | 0.995 - 0.998 | <0.0001 |
| **Glutamate at 24 h** | 0.99 | 0.99 - 0.99 | <0.0001 | 0.993 | 0.990 - 0.996 | <0.0001 |
|  |  |  |  |  |  |  |
| **IL6 on admission** | 1.00 | 0.99 - 1.01 | 0.292 | - | - | - |
| **IL6 at 24 h** | 0.99 | 0.99 - 0.99 | 0.019 | 0.990 | 0.985 - 0.995 | <0.0001 |

** Unadjusted model.*

*** Adjusted by: age, atrial fibrillation, temperature, glycemia, leukocytes, fibrinogen, NIHSS on admission, infarct volume, study group and onset time.*

Table S5 Logistic regression analysis for extra-hospital improvement related with Glutamate and IL6 serum levels on admission and at 24 hours.

|  | **OR*** | **CI 95%** | **p** | **OR**** | **CI 95%** | **p** |
| --- | --- | --- | --- | --- | --- | --- |
| **Glutamate on admission** | 1.00 | 1.00 - 1.00 | <0.0001 | 1.001 | 1.001-1.003 | 0.113 |
| **Glutamate at 24 h** | 1.10 | 1.07 - 1.12 | <0.0001 | 1.132 | 1.072 - 1.196 | 0.001 |
|  |  |  |  |  |  |  |
| **IL6 on admission** | 0.99 | 0.98 - 1.01 | 0.520 | - | - | - |
| **IL6 at 24 h** | 1.10 | 1.08 - 1.11 | <0.0001 | 1.137 | 1.094 - 1.183 | <0.0001 |

** Unadjusted model.*

*** Adjusted by: age, atrial fibrillation, temperature, glycemia, leukocytes, fibrinogen, NIHSS on admission, infarct volume, study group and onset time.*

**Table S6**. Multiple Linear Regression Analysis for intra and extrahospital improvement. Correlation with glutamate and IL6 levels on admission and after 24 hours.

| ***Intrahospital Improvement*** | | | | | | | | | |
| --- | --- | --- | --- | --- | --- | --- | --- | --- | --- |
| **Independent variables** | **β** | **CI 95%** | | **p** |  | **β *** | **CI 95%** | | **p** |
| **Glutamate on admission (uM)** | 0.003 | -0.004 | 0.011 | 0.347 |  | 0.025 | 0.004 | 0.045 | 0.017 |
| **Glutamate 24h (uM)** | -0.072 | -0.083 | -0.060 | <0.0001 |  | -0.042 | -0.083 | -0.001 | 0.046 |
|  |  |  |  |  |  |  |  |  |  |
| **IL6 on admission (pg/mL)** | 0.034 | -0.025 | 0.092 | 0.256 |  | 0.082 | -0.109 | 0.274 | 0.397 |
| **IL6 24 h(pg/mL)** | -0.081 | -0.103 | -0.059 | <0.0001 |  | -0.121 | -0.203 | -0.038 | 0.004 |
| ***Extrahospital Improvement*** | | | | | | | | | |
| **Independent variables** | **β** | **CI 95%** | | **p** |  | **β **** | **CI 95%** | | **p** |
| **Glutamate on admission (uM)** | 0.018 | 0.013 | 0.023 | <0.0001 |  | 0.019 | 0.007 | 0.031 | 0.002 |
| **Glutamate 24h (uM)** | 0.067 | 0.060 | 0.074 | <0.0001 |  | 0.015 | -0.008 | 0.038 | 0.202 |
|  |  |  |  |  |  |  |  |  |  |
| **IL6 on admission (pg/mL)** | -0.015 | -0.058 | 0.028 | 0.483 |  | -0.305 | -0.405 | -0.204 | <0.0001 |
| **IL6 24 h(pg/mL)** | 0.121 | 0.109 | 0.134 | <0.0001 |  | 0.131 | 0.086 | 0.176 | <0.0001 |

*Adjusted by: Onset-Inclusion, Age, Sex, temperature, Glycemia, Hemorrhagic transformation, TOAST, Study group

**Adjusted by: Onset-Inclusion, Age, History of enolism, History of atrial fibrilation, Previous TIA, Glycemia, Leukocytes, Fibrinogen, Reactive protein C, Triglycerides, Sedimentation Rate, ProBNP, NIHSS on admission
